# Bilateral breast metastases from small cell lung carcinoma: Case report and review of the literature

**DOI:** 10.1016/j.radcr.2021.03.056

**Published:** 2021-04-30

**Authors:** Shuchi Zinzuwadia, John Olivieri, Cheryl Zhang, Vijayalakshmi Ananthanarayanan, Luke Freiburg, Emad Allam

**Affiliations:** Loyola University Medical Center, Department of Radiology, 2160 S 1st Ave, Maywood, IL 60153 USA

**Keywords:** Small cell lung cancer, Neuroendocrine, Breast metastases, Secondary breast cancer, ER, estrogen receptor, PR, progesterone receptor, HER2, human epidermal growth factor receptor 2, CD45, cluster of differentiation 45, LCA, leukocyte common antigen, CDX-2, caudal type homeobox 2, Ki-67, proliferation index, TTF-1, thyroid transcription factor1, PD-L1, programmed death-ligand 1, BI-RADS, breast imaging reporting and data system, WHO, world health organization

## Abstract

Differentiation of primary versus secondary breast cancer can be difficult, with the relative rarity of the latter representing a diagnostic challenge. Here, we present a case of small cell lung cancer with synchronous bilateral breast metastases in a 52-year-old female. There are less than 5 other cases of small cell lung cancer with bilateral breast metastases reported in the literature to date. The breast metastases represented the first clinical and imaging manifestation of malignancy in our case. We present the patient's disease course including multi-modal imaging, histopathologic analysis, and clinical management. We aim to highlight the entity of secondary breast cancer and how multidisciplinary collaboration can help arrive at the diagnosis, which is critical for prognosis and treatment planning in this patient population.

## Background

Metastases to the breast from an extramammary malignancy are rare compared to primary breast cancer, comprising only 0.4%- to 1.3% of all cancers found in the breast [1]. Accurate diagnosis is important for appropriate patient management, particularly since surgical intervention is typically not performed for metastatic disease [2]. A wide variety of extramammary malignancies have been reported to metastasize to the breast, with hematopoietic malignancies being the most common [3]. Metastasis of small cell lung cancer to the breast is extremely rare. We present a case of synchronous bilateral breast metastases from small cell lung cancer. The breast masses were the first manifestation of malignancy in this patient.

**Fig. 1:**
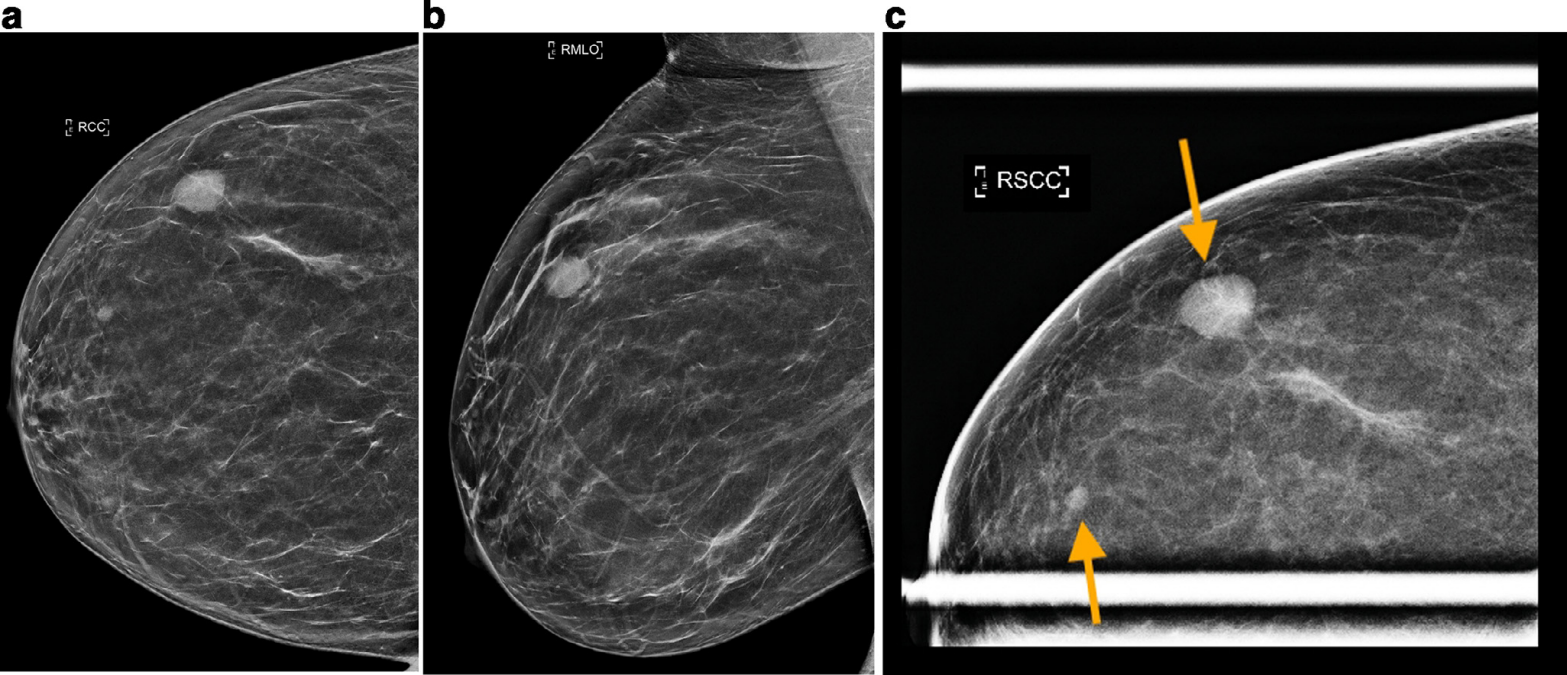
Diagnostic mammography was obtained utilizing tomosynthesis with reconstructed 2D composite views (C-views). Right breast full field (a) CC and (b) MLO C-views, and (c) conventional 2D spot compression CC view demonstrate two high density masses in the right upper outer breast (indicated by the arrows on the spot compression view). This included the patient-reported palpable, oval, circumscribed 1.5 cm mass at the 11-o'clock position, as well as a 0.4 cm oval but indistinctly marginated mass at the 10-o'clock position. These were new since prior screening mammogram 1.5 years ago, and findings were classified as BI-RADS 4.

**Fig. 2:**
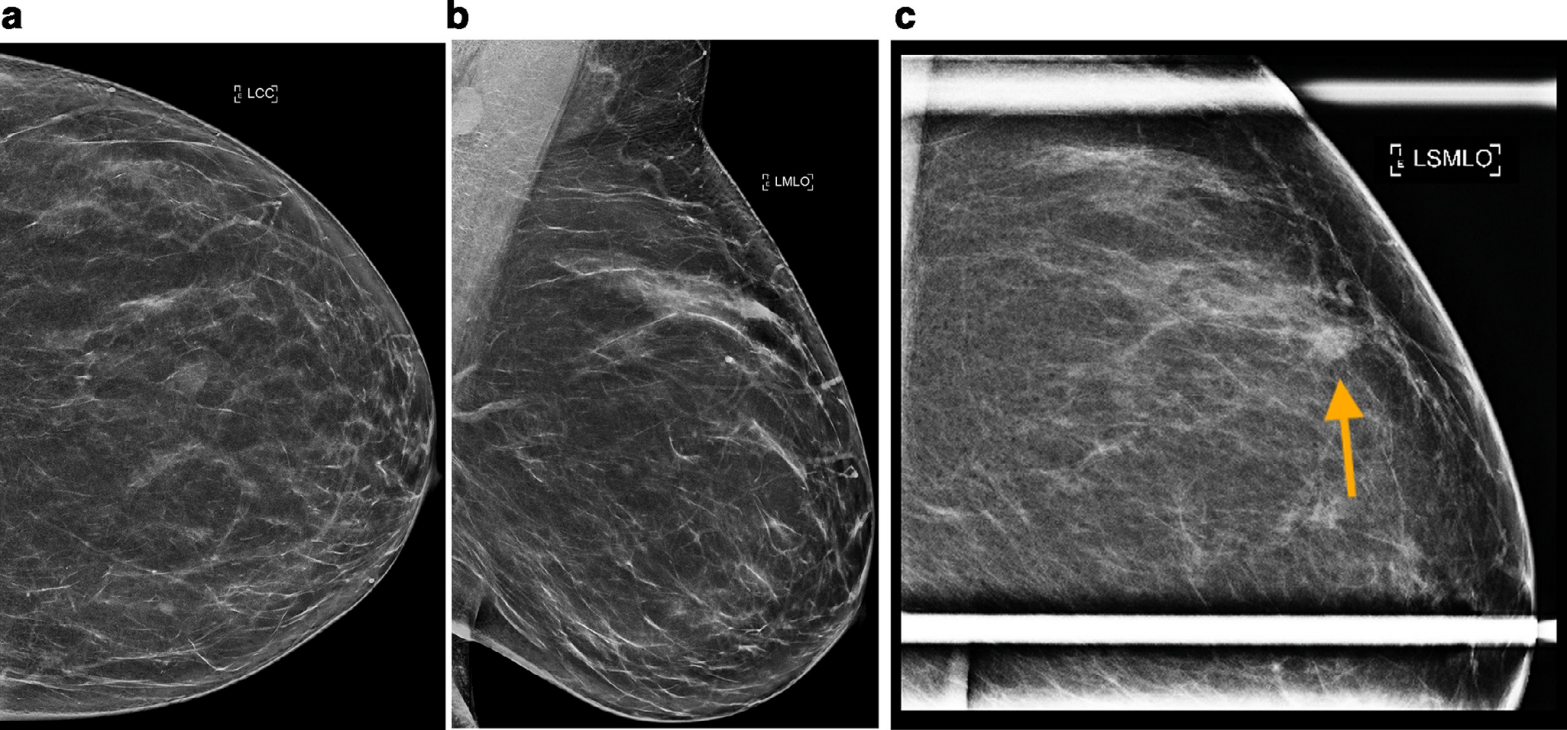
Diagnostic mammography was obtained utilizing tomosynthesis with reconstructed 2D composite views (C-views). Left breast full field (a) CC and (b) MLO views, and (c) conventional 2D spot compression MLO view demonstrate a 1.0 cm indistinctly marginated high density mass in the left upper breast at the 12-o'clock position (indicated by the arrow on the spot compression view). This was new since prior screening mammogram 1.5 years ago, and findings were classified as BI-RADS 4.

**Fig. 3:**
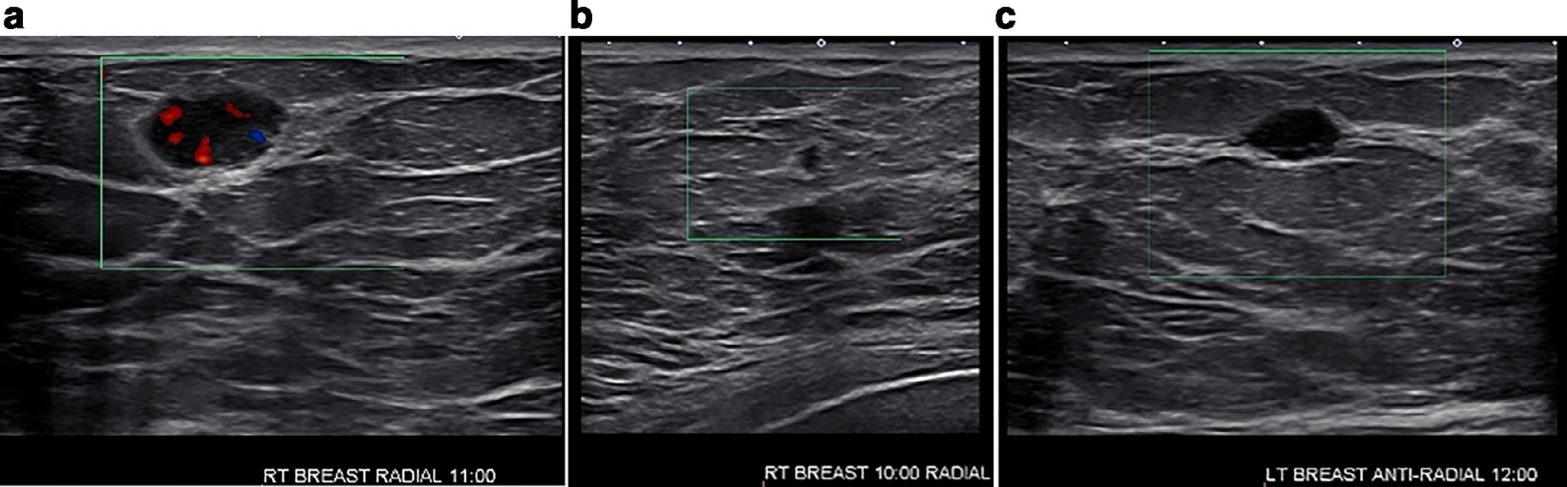
Diagnostic high resolution sonography of both breasts confirmed ultrasound correlates for the mammographic masses, all classified as BI-RADS 4. This included (a) targeted sonography with color Doppler demonstrating an oval, circumscribed, parallel oriented, hypoechoic 1.5 cm right breast mass with internal vascularity at 11-o'clock, 9 cm from the nipple, corresponding to the patient's palpable complaint, (b) an irregular, spiculated 0.4 cm right breast mass with anti–parallel orientation at 10-o'clock, 10 cm from the nipple, and (c) an oval, circumscribed, parallel oriented, hypoechoic 1.0 cm left breast mass at 12-o'clock, 6 cm from the nipple.

## Case presentation

### Initial presentation and imaging

A 52-year-old woman with no history of smoking or history of malignancy presented with bilateral breast tenderness and a palpable mass in her right outer breast. Screening mammograms performed 1.5 years prior to the development of symptoms were negative. Diagnostic mammograms were obtained to evaluate the breast symptomatology and demonstrated 2 new masses in the right breast and 1 in the left (Fig. 1 and 2). Note that the largest/palpable mass appeared oval in shape with circumscribed margins. Since these masses were new from the prior study, further evaluation with ultrasound was performed. Targeted breast ultrasound confirmed indeterminate imaging features of the breast masses (Fig. 3), and findings were all classified as BI-RADS 4, suspicious. Ultrasound-guided core needle biopsies of all 3 breast masses were performed at an outside institution. Histopathologic examination of all 3 masses was consistent with high grade neuroendocrine neoplasm. No ductal carcinoma in situ was observed. Breast markers including ER, PR, and HER2 were negative. Synaptophysin and chromogranin were diffusely positive in the tumor cells, supportive of neuroendocrine origin. Immunohistochemistry was negative for pankeratin, cytokeratin 7, cytokeratin 20, GATA-3, and CD45 (LCA). TTF-1 positivity suggested the possibility of a lung primary, and absence of CDX-2 made a gastrointestinal primary less likely [4]. Ki-67 expression was reported as high as 70%.

**Fig. 4:**
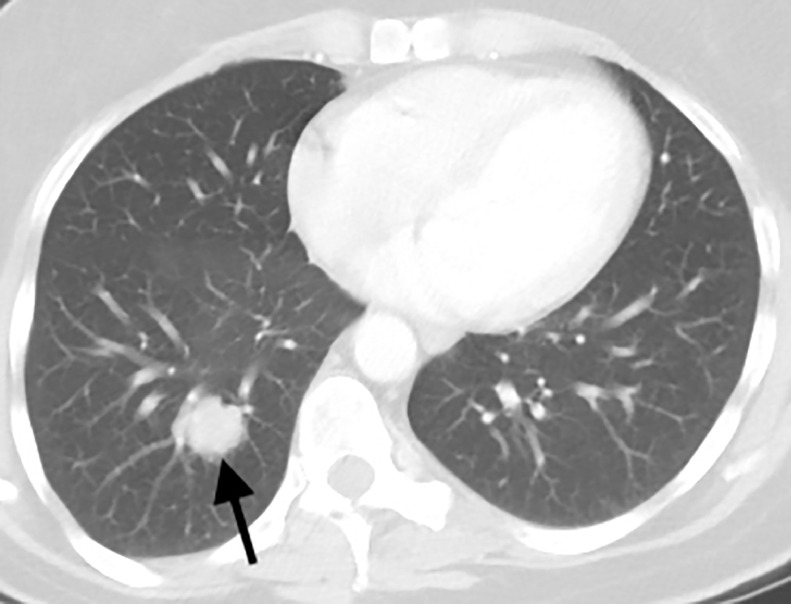
CT demonstrating a 2.2 cm right lower lobe pulmonary nodule abutting the bronchovascular bundle (arrow).

**Fig. 5:**
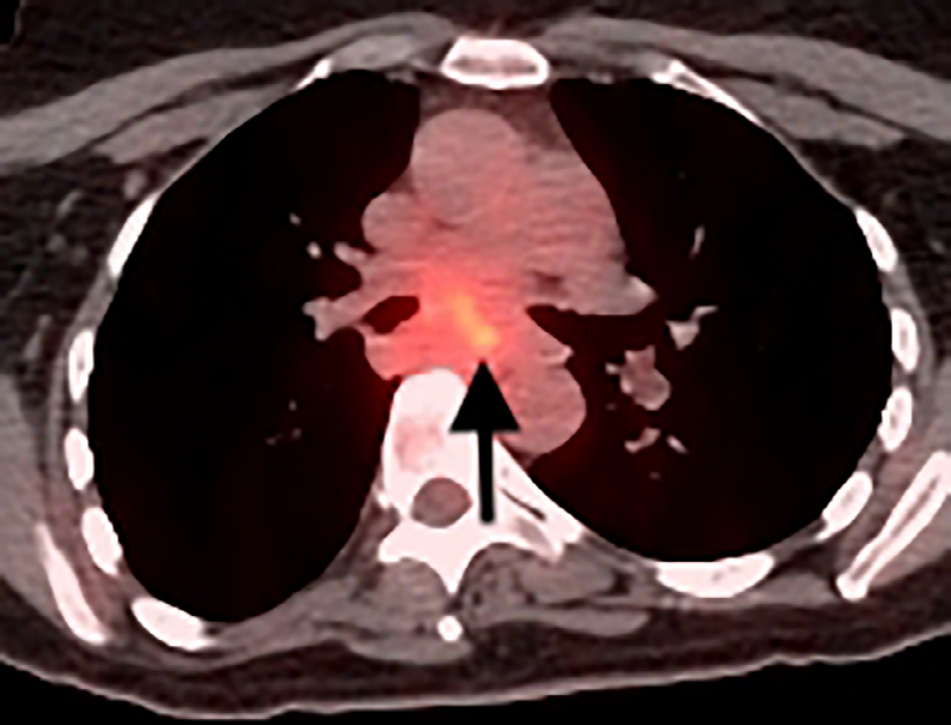
PET/CT demonstrating FDG-avid subcarinal lymphadenopathy (arrow).

**Fig. 6:**
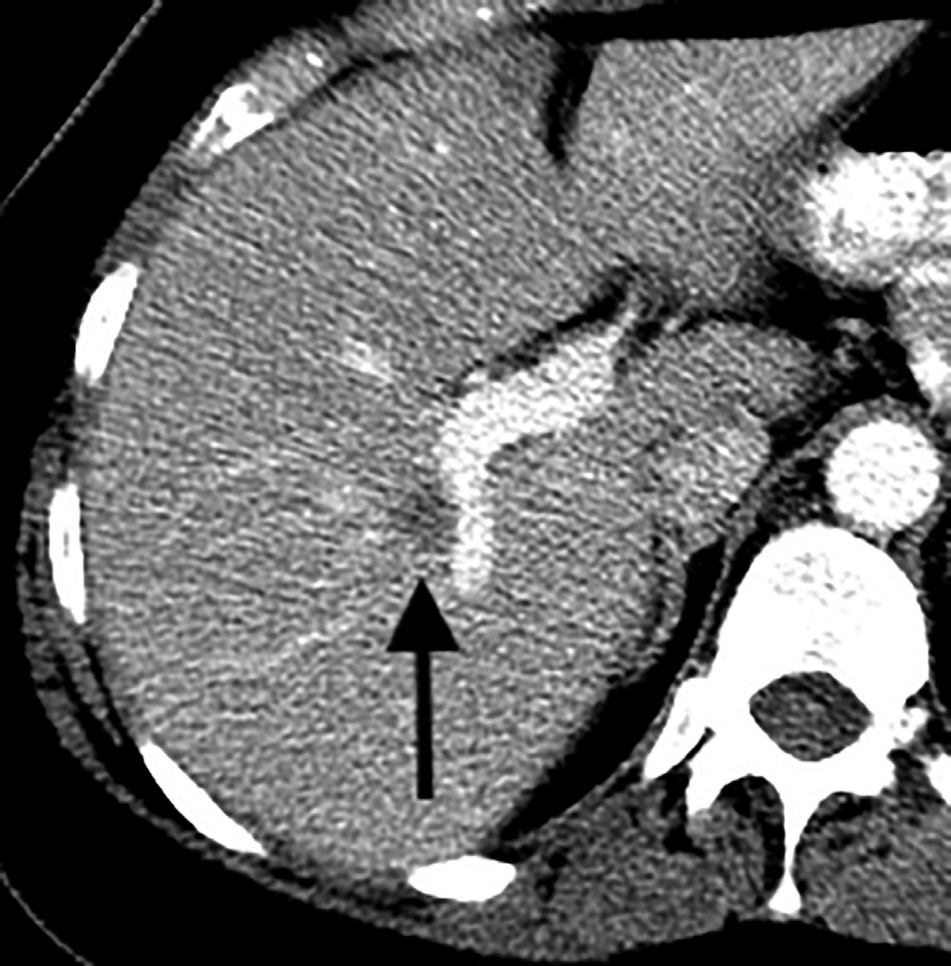
Contrast-enhanced CT demonstrating a 1.1 cm hypoattenuating lesion in the right hepatic lobe abutting a branch of the right portal vein (arrow).

**Fig. 7:**
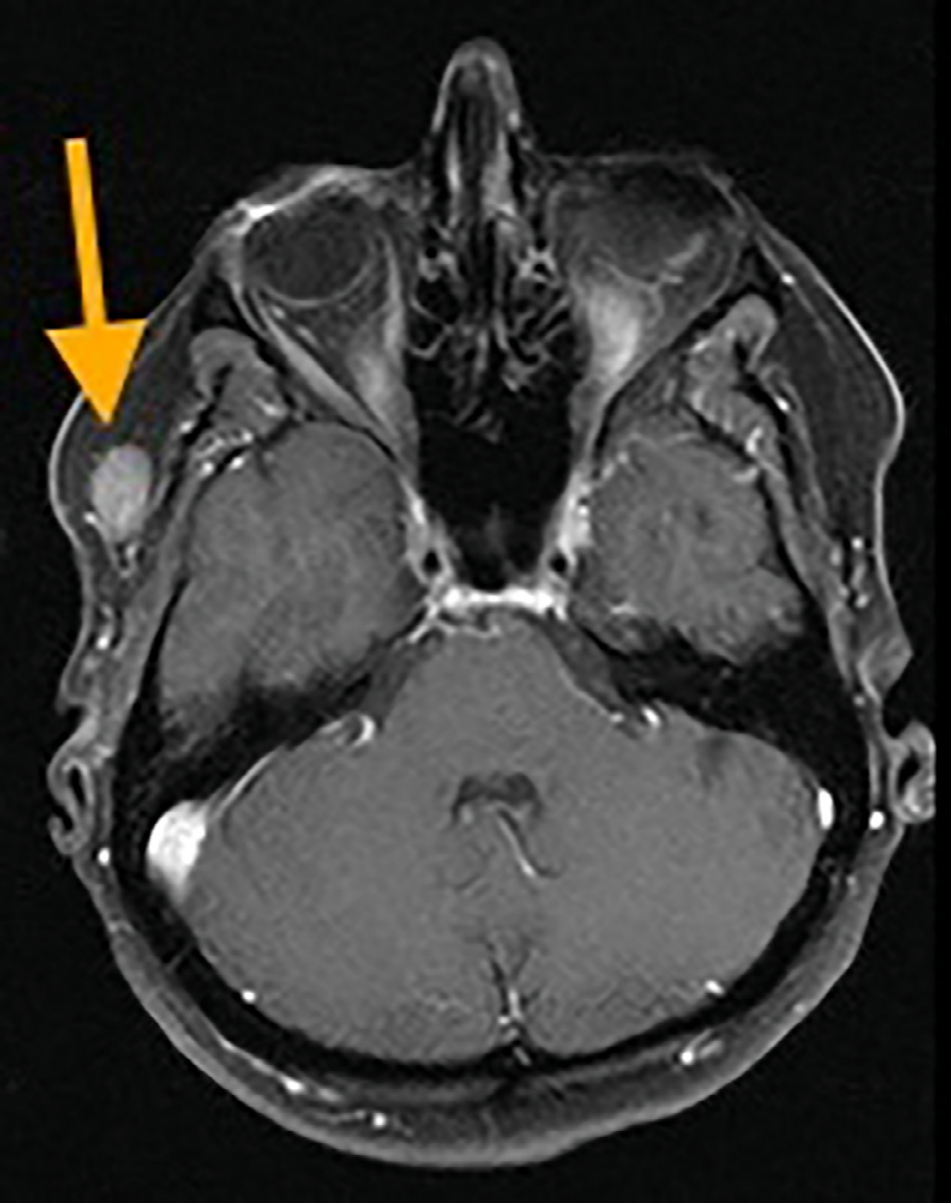
T1-weighted fat-saturated post-contrast MRI sequence demonstrating a 1.6 cm enhancing lesion in the right temporal subcutaneous tissues (arrow). There is normal enhancement of the right sigmoid sinus. No intracranial lesion was identified.

CT of the chest, abdomen, and pelvis, PET/CT, and MRI of the brain were performed for initial staging. In addition to the biopsied bilateral breast masses, imaging demonstrated a 2.2 cm right lower lobe pulmonary nodule in a central location (Fig. 4), ipsilateral hilar and mediastinal lymphadenopathy (Fig. 5), a single 1.1 cm liver lesion (Fig. 6), and a nodule in the right temporal subcutaneous tissues (Fig. 7). No axillary lymphadenopathy was noted. Based on imaging and pathology, this was determined to be a primary lung small cell carcinoma with metastases, including bilateral breast metastases. This was staged as T1c N2 M1.

## Treatment and follow up imaging

**Fig. 8:**
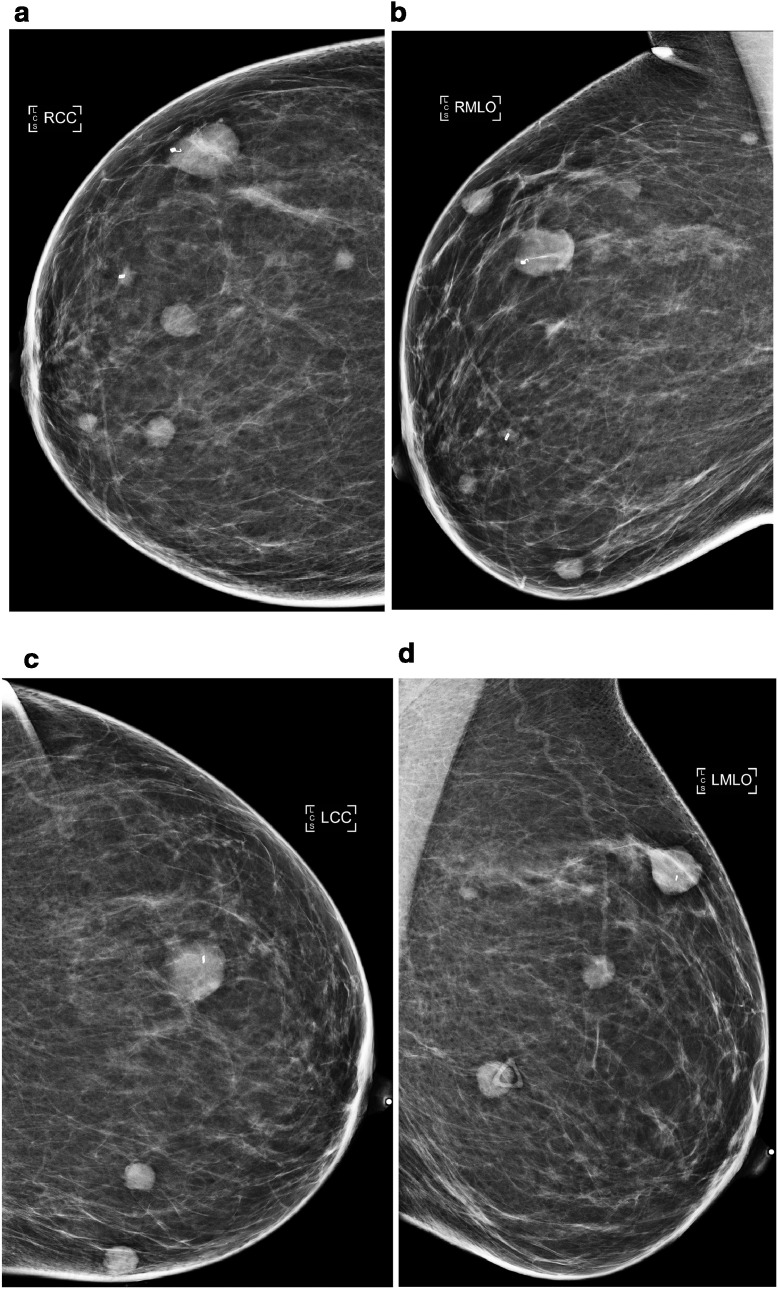
Diagnostic 2D full field CC and MLO mammograms of the right breast (a and b) and left breast (c and d) obtained 10 months after the initial diagnostic mammograms demonstrate interval increase in size of all biopsy proven bilateral metastatic sites (now containing biopsy clips), with representative palpable right breast mass now measuring 2.0 cm (previously 1.5 cm). There are 5 right and 3 left new, oval, circumscribed, high density masses. Findings were deemed compatible with disease progression and classified as BI-RADS 6, known biopsy-proven malignancy. Compare to prior diagnostic mammograms in Fig. 1 and 2.

**Fig. 9:**
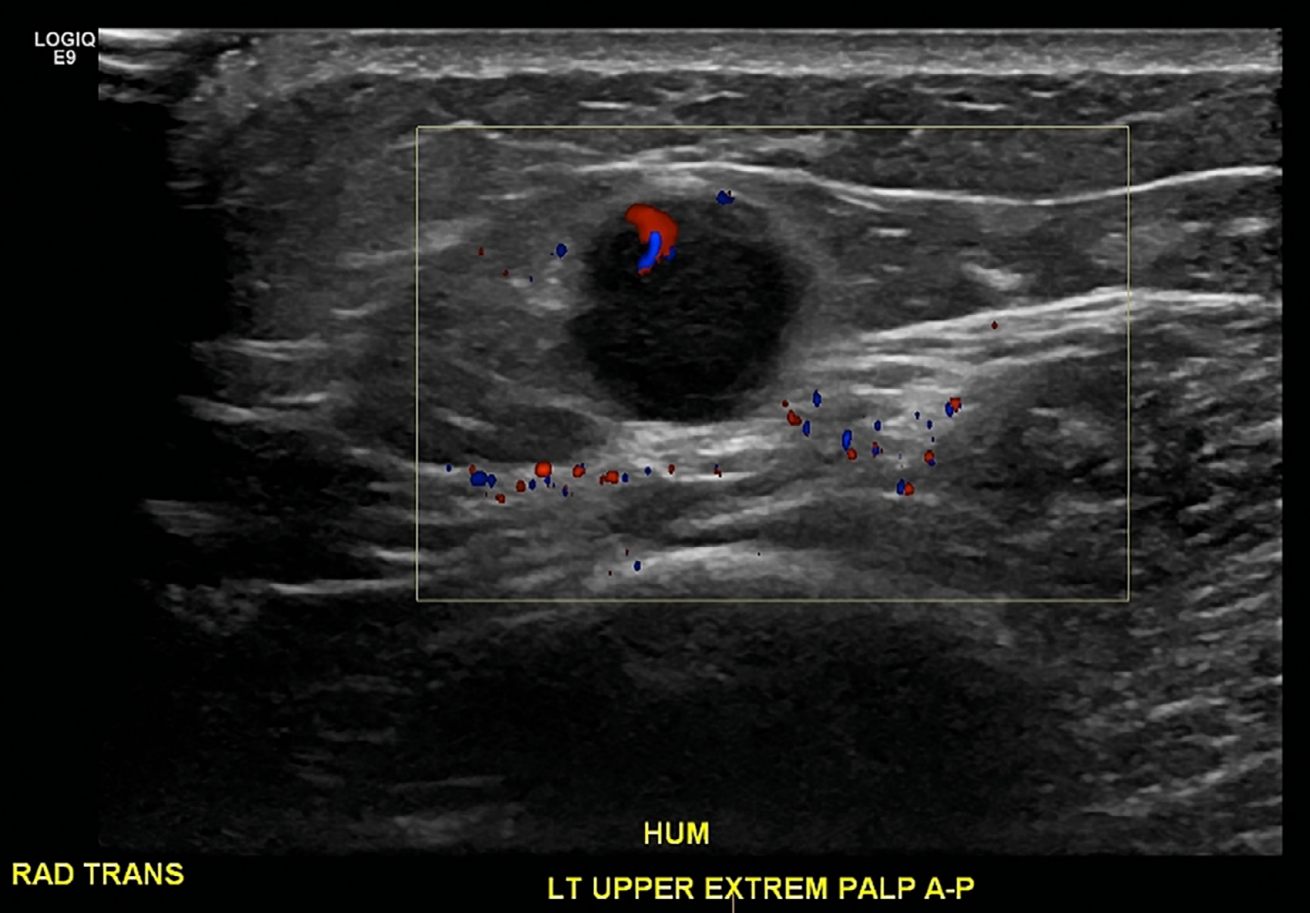
Targeted ultrasound of the left upper extremity at the level of the distal humerus demonstrates a 1.3 cm round hypoechoic lesion in the soft tissues with internal vascularity, corresponding to the palpable abnormality. The lesion is suspicious for metastasis, with an epitrochlear lymph node disfavored given the lateral location.

**Fig. 10:**
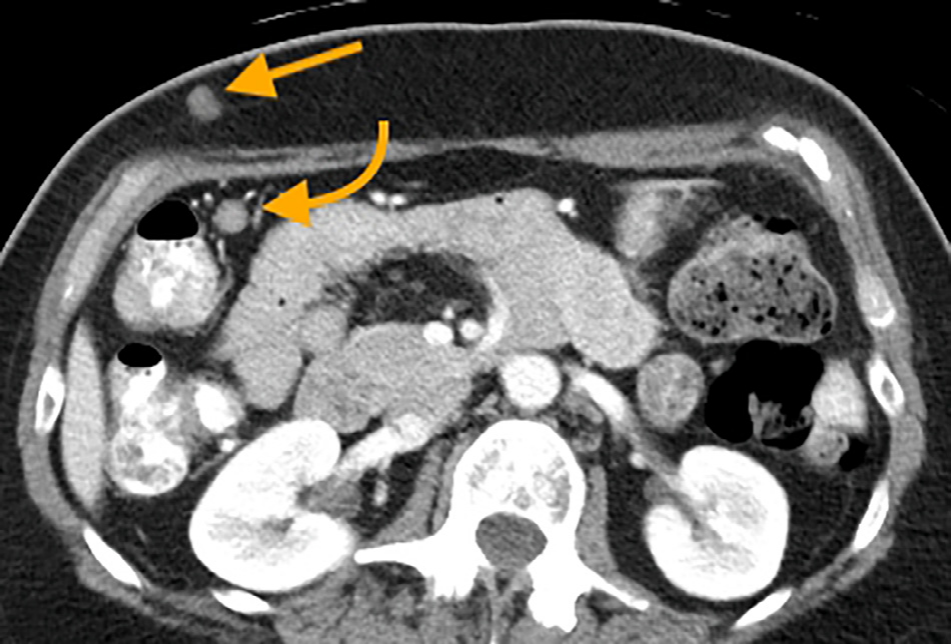
CT demonstrating a subcutaneous nodule in the right abdominal wall (straight arrow) and a prominent mesenteric lymph node in the right abdomen (curved arrow).

The patient started first line chemotherapy 2 months after the initial diagnostic mammograms and completed 4 cycles of carboplatin/etoposide/atezolizumab. Subsequently, she was transitioned to maintenance atezolizumab. However, the patient reported new palpable and/or tender nodules in the bilateral breasts, left arm, and abdominal wall. Imaging demonstrated overall progression of metastatic disease despite chemotherapy. In particular, diagnostic mammograms obtained 10 months after the initial diagnostic mammograms demonstrated interval increase in size and number of bilateral breast masses (Fig. 8). Ultrasound demonstrated a 1.3 cm hypoechoic mass in the soft tissues over the left distal humerus suspicious for metastatic disease (Fig. 9). CT demonstrated interval development of subcutaneous abdominal wall nodules measuring up to 1.1 cm and a new 0.9 cm mesenteric lymph node (Fig. 10).

**Fig. 11:**
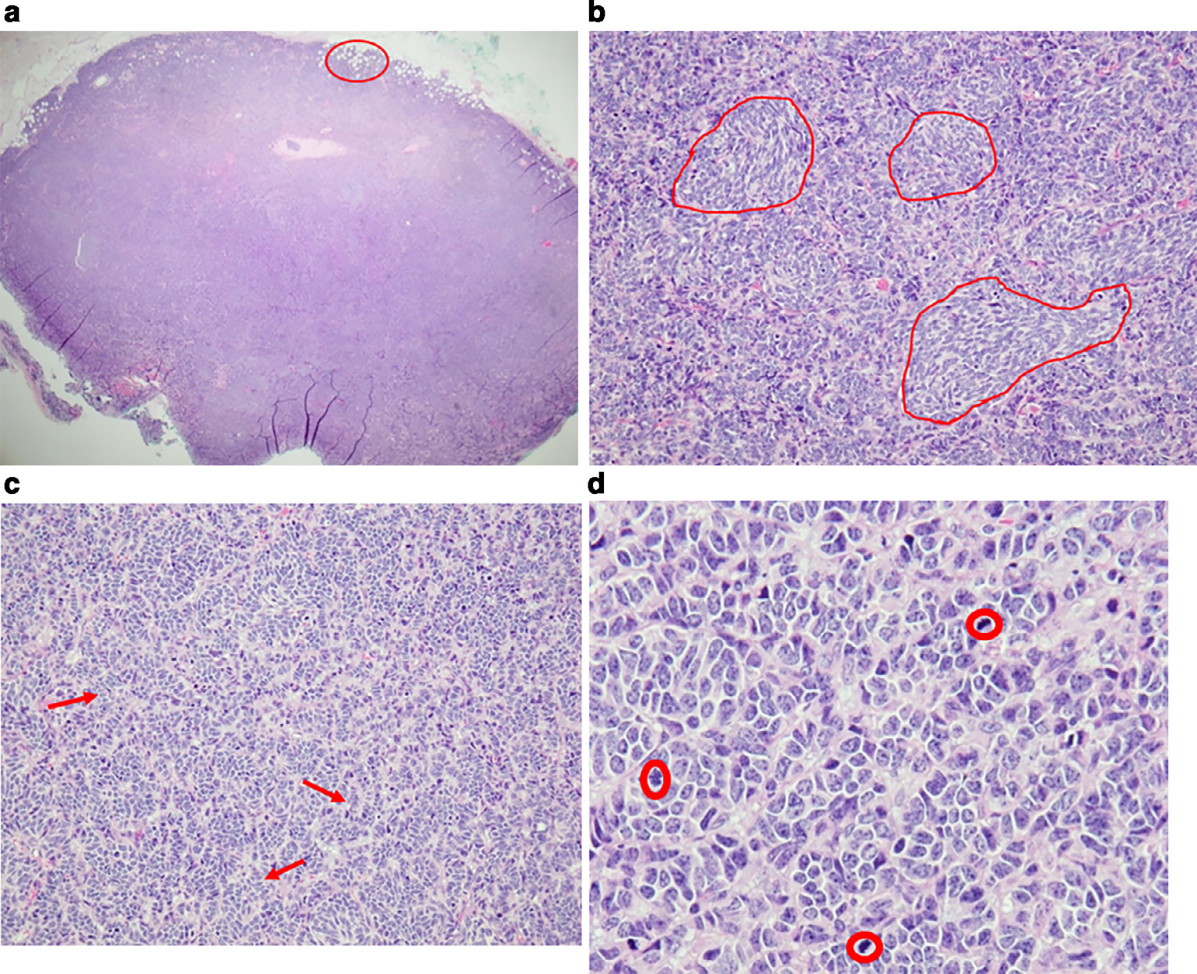
Histology of the excised subcutaneous abdominal wall nodule consistent with metastatic small cell carcinoma. (a) Low power magnification (4x) shows a sharply demarcated subcutaneous nodule of basaloid tumor cells. Tumor cell infiltration into the surrounding adipose tissue is noted (circle). (b) Spindly to ovoid basaloid tumor cells arranged in nests (loops). (c) Tumor cells amidst infiltrating cords and trabeculae (arrows). (d) Tumor cells with high nuclear to cytoplasmic ratio, hyperchromatic nuclei and brisk mitotic activity (circles).

**Fig. 12:**
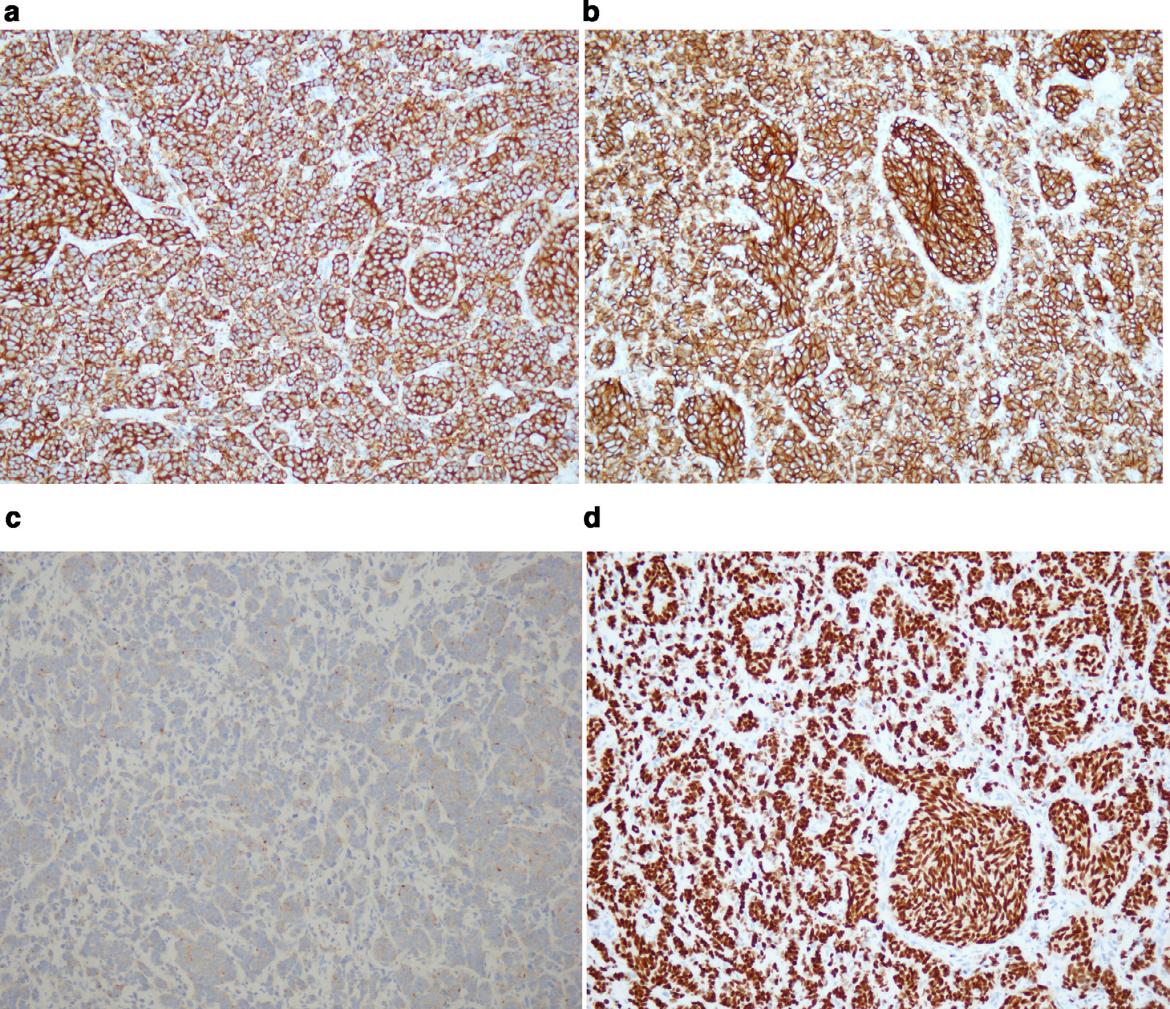
Immunohistochemistry of the excised subcutaneous abdominal wall nodule consistent with metastatic small cell carcinoma. (a and b) Tumor cells are strongly and diffusely positive for neuroendocrine markers, particularly (a) synaptophysin and (b) CD56. (c) The tumor is also positive for chromogranin, a more specific neuroendocrine marker, albeit weakly and with a dot-like staining pattern. (d) TTF-1 labels nearly 100% of tumor nuclei. Diffuse and strong TTF-1 immunostaining is noted in the vast majority of pulmonary small cell carcinomas, although a proportion of extrapulmonary small cell carcinomas can also express TTF-1, depending on the clone of antibody used [5].

Excisional biopsy of one of the abdominal wall nodules was performed. Pathology of the abdominal wall nodule was consistent with metastatic small cell carcinoma with similar findings as the breast masses, except for a relatively reduced Ki-67 index of 30% to 35% possibly reflecting chemotherapy related changes (Fig. 11 and 12). No PD-L1 expression was detected, precluding treatment with PD-L1 inhibitors. Given the rapid progression of disease, chemotherapy was changed to lurbinectedin, a second line agent for patients with metastatic small cell cancer demonstrating disease progression on platinum-based chemotherapy. However, follow-up imaging showed continued progression of metastatic disease even with second line chemotherapy.

## Discussion

Metastatic cancer to the breast is uncommon [2]. The incidence of metastatic disease to the breast may be increasing due to improved survival of patients with malignancy [6]. The most common metastasis is spread from a contralateral breast carcinoma. Lymphoma, leukemia, and melanoma are common malignancies that may involve/metastasize to the breast [7]. Lung cancer is one of the most common cancers in terms of overall incidence, but rarely metastasizes to the breast [8,9]. Of the lung cancers metastasizing to the breast, most are adenocarcinoma. Other types of lung cancer, including small cell carcinoma, seldomly metastasize to the breast [10]. It is possible that secondary breast cancers may be more frequent than previously thought, as some may have been misdiagnosed as primary tumors [11]. Increased awareness, modern technology and imaging tools may help in correct diagnosis.

Four major types of lung neuroendocrine neoplasms are recognized by the WHO: typical carcinoid, atypical carcinoid, small cell lung cancer, and large cell neuroendocrine carcinoma [11]. There have been 2 prior case reports of neuroendocrine neoplasm of unspecified type with bilateral breast metastases [1,6], and only 1 other documented case of small cell lung carcinoma with bilateral breast metastases [12]. Bilateral breast metastases from an atypical lung carcinoid has also been reported [11]. Note that primary mammary small cell carcinoma is very rare. The presence of ductal carcinoma in situ or estrogen receptor positivity would favor a diagnosis of primary mammary small cell carcinoma [3]. Hoang et al. showed that primary breast small cell carcinoma is clonally related to ductal carcinoma in situ and might represent an instance of divergent differentiation occurring in a multipotent neoplastic stem cell [13].

A palpable breast mass may be the reason for initial presentation in patients with metastatic disease, as was seen in our case. In a review by Mirrielees et al., the majority of small cell lung cancer metastases to the breast were classified as synchronous ie presenting clinically at the same time as the primary lung carcinoma rather than later [14]. Synchronous presentation can make it difficult to differentiate between primary and secondary breast cancer. In our case, the patient was a non–smoker and did not have significant pulmonary symptoms, and therefore lung cancer was not suspected clinically. Mammography and sonography usually reveal round or oval circumscribed masses; calcification and spiculation seen with primary breast carcinomas are uncommon in secondary breast cancers [2]. Unlike primary breast cancers which have proliferation of fibrous connective tissue around the tumor, metastatic lesions lack this desmoplastic response and associated architectural distortion [14]. Thus, metastatic breast masses may be mistaken for benign lesions such as fibroadenomas on imaging due to their circumscribed appearance and multiplicity. Metastatic lesions are more likely to be superficial in location compared to primary breast cancers, yet do not demonstrate skin or nipple retraction [8,14].

The diagnosis of metastatic small cell carcinoma to the breast may be challenging on pathology, and a panel of immunohistochemical stains is often required. TTF-1 is almost exclusively expressed during thyroid and lung organogenesis and is a proposed biomarker of primary lung carcinoma [14]. The distribution of disease on imaging may aid in diagnosis. It is important to distinguish primary small cell carcinoma of the breast from metastatic small cell carcinoma of the lung, since the former is typically treated surgically while the latter is typically treated with chemotherapy. A slightly better prognosis is seen in patients with primary small cell carcinoma of the breast relative to metastatic small carcinoma to the breast, although the rarity of the condition makes it difficult to predict prognosis [6,10].

## Conclusion

Although rare, metastatic disease to the breast should be considered in the context of breast masses detected on physical examination or imaging. These masses may have a benign appearance on imaging. Differentiation of primary breast cancer from secondary breast cancer is critical for management and prognosis, and imaging and immunohistochemistry are helpful for achieving the correct diagnosis. These metastases may be synchronous with the primary tumor and thus the patient may have no known history of malignancy. Close imaging follow up is advised for these rare cases as patients may not respond to standard chemotherapeutic agents, as illustrated in this case.
